# Talocrural Arthrodesis Increases Osteoarthritis Severity in Adjacent Joints: A Midterm Computed Tomography Follow-Up Study

**DOI:** 10.1177/19386400231208533

**Published:** 2023-11-02

**Authors:** Annika Willems, Mauro Minnaard, Edwin H.G. Oei, Sita M.A. Bierma-Zeinstra, Duncan E. Meuffels

**Affiliations:** Department of Orthopaedics and Sports Medicine; Department of Orthopaedics and Sports Medicine; Department of Radiology and Nuclear Medicine; Department of General Practice, Erasmus MC University Medical Centre, Rotterdam, The Netherlands; Department of Orthopaedics and Sports Medicine

**Keywords:** foot, talocrural, arthrodesis, osteoarthritis, computed tomography

## Abstract

**Background:**

After talocrural arthrodesis, adjacent joints (subtalar, talonavicular, and calcaneocuboid) are often affected by osteoarthritis (OA)). It is unclear if OA is pre-existing to talocrural arthrodesis, or whether it develops after talocrural arthrodesis. This retrospective study is unique because it is the first study with preoperative and follow-up computed tomography (CT). The aim of this study is to investigate whether OA develops in adjacent joints after talocrural arthrodesis or if OA is already pre-existing. In addition, associations between degree of OA and patient-reported outcomes are investigated.

**Methods:**

Patients were selected from electronic files, and adjacent joint OA was assessed on preoperative CT and bilateral follow-up CT. Patient-reported outcomes were collected.

**Results:**

Twenty-three patients were included with an average follow-up time of 7 years (SD = 2). In participants without pre-existing OA, OA significantly progressed in all adjacent joints. In participants with pre-existing OA, OA progressed in the subtalar joint. Patient-reported outcomes were not correlated to OA.

**Conclusions:**

Osteoarthritis in the adjacent joints progresses after talocrural arthrodesis, especially in participants without pre-existing OA. The severity of OA is not related to patient-reported outcomes. Therefore, the clinical impact of the progression of OA seems to be limited.

**Level of Evidence:**

Level III: retrospective

“Osteoarthritis in adjacent joints progresses after talocrural arthrodesis, but the clinical impact of osteoarthritis progression seems to be limited.”

## Introduction

A talocrural arthrodesis can be a life-changing operative intervention for patients with end-stage talocrural osteoarthritis (OA). After talocrural arthrodesis, pain scores are significantly reduced resulting in a better quality of life.^1^ However, mid- and long-term follow-up studies showed that OA is present in adjacent joints (subtalar, talonavicular, and calcaneocuboid joints) after talocrural arthrodesis.^2^ It has been hypothesized that these arthritic changes develop after talocrural arthrodesis as a consequence of increased use and higher forces in the joints.^3,4^ In contrast, it has been suggested that arthritic changes are pre-existing to talocrural arthrodesis.^5^ Although adjacent joint OA after arthrodesis is a widely studied subject,^6-11^ a paucity of studies evaluate the preoperative prevalence of adjacent joint OA.^2^ It is therefore unclear whether adjacent joint OA is present before talocrural arthrodesis or if it develops after talocrural arthrodesis.^2^

Also, so far, all studies describing adjacent joint OA after talocrural arthrodesis have used radiographs to assess OA^2,6-11^. The assessment of OA in the subtalar and talonavicular joints from radiographs with Kellgren-Lawrence score showed poor reliability, as radiographs lack bony details for reliable OA assessment.^12,13^ Alternatively, computed tomography (CT) provides cross-sectional images, from which all parts of the joints can be assessed in detail and are more accurate.^14^

This study presents a cohort of patients who underwent preoperative and postoperative CT. It is unique because both preoperative and postoperative CT is available, thus development or progression of OA in adjacent joints can be precisely determined. We aim to assess whether adjacent joint OA is present before talocrural arthrodesis, or if it develops postoperatively. Furthermore, we will also correlate OA in adjacent joints to the length of follow-up, patient-reported outcome measures, and measures of patients’ satisfaction.

## Methods

### Study Design and Participants

**Figure 1.**
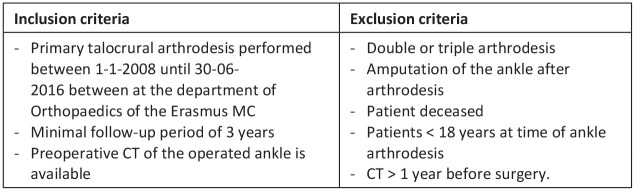
Study inclusion and exclusion criteria. Abbreviation: CT, computed tomography.

This retrospective cohort presents data of patients who underwent talocrural arthrodesis at the Erasmus MC University Medical Center, Rotterdam, the Netherlands between January 2008 and June 2016. Patients were indicated for an isolated talocrural arthrodesis if they experienced symptomatic talocrural OA, sometimes accompanied with postural deviations of the ankle, and without complaints of the adjacent joints and postural deviations in the foot. An electronic search in the hospital files was performed based on operative codes, to select all patients who underwent a talocrural arthrodesis. After the electronic search, patients were screened for eligibility based on predefined inclusion and exclusion criteria (Figure 1). Eligible patients were approached to participate in the study.

Patients who were willing to participate gave written informed consent and were invited to the outpatient clinic for follow-up examination. Follow-up examination included bilateral CT and completion of patient-reported outcome measures.

### Outcome Measures

#### Baseline characteristics

Baseline characteristics were extracted from the electronic patient file (age, body mass index [BMI], sex, operated side, reason for talocrural arthrodesis, technique used for talocrural arthrodesis, and date of talocrural arthrodesis). At follow-up examination, participants were asked whether they experienced problems with the contralateral ankle.

#### Grading OA of tarsal joints with CT OA scale

**Figure 2.**
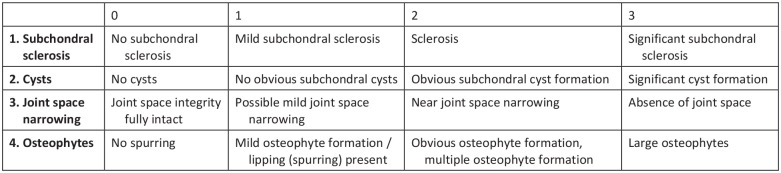
CT OA scale: scoring tool for OA in ankle and foot joints from CT. Abbreviations: CT, computed tomography; OA, osteoarthritis.

The degree of OA in the adjacent joints was assessed on the preoperative and on bilateral follow-up CT, where the non-affected ankle served as a control. The degree of OA in the adjacent joints was assessed with a modified assessment tool based on the Kellgren-Lawrence OA scoring (0-4) and CT ankle OA atlas.^14,15^ This modified tool, the CT OA scale, contains 4 features which are associated with OA: subchondral sclerosis, cysts, joint space narrowing and osteophytes. Each feature was scored separately on a scale from 0 to 3, where 0 is absence of the feature and 3 the worst severity of the feature. Figure 2 shows the grading of the CT OA scale. After all features were scored, a total score per joint was calculated by totalling all scores of the individual features. The total score ranges between 0 representing no OA, and 12 representing worst OA. Joints were assessed based on multiplanar reconstructions in 3 planes (sagittal, coronal, and axial), with slice thickness of 1 mm. All CT’s were scored by one observer (AW). A random sample of 12 CT’s was scored by a second observer (DM) to assess interrater reliability for each feature and for the overall score.

After scoring CT’s with CT OA scale, observers were asked to indicate whether, in their opinion, OA was present in the joint or not (yes or no). These outcomes were used to set a cut-off value for the OA ankle scale to discriminate between joints with pre-existing OA and joints without pre-existing OA.

#### Patient-reported outcome measures

Patient-reported outcome measures were assessed with 3 questionnaires. The “36-Item Short Form Health Survey” (SF-36) about quality of life, containing 8 subdomains (physical functioning, limitations due to physical health, limitations due to emotional problems, energy/fatigue, emotional well-being, social functioning, pain, and general health).^16^ The Foot and Ankle Outcome Score (FAOS) assesses ankle function and pain.^17^ The American Orthopaedic Foot and Ankle Society (AOFAS) Ankle-Hindfoot scale assesses pain and impairment of the ankle.^18^ The AOFAS Ankle-Hindfoot scale has a clinical reported part also, which was completed by the Orthopaedic surgeon in residence. The scores of all 3 patient-reported outcome measures range between 0 and 100, where a higher score indicates better quality of life (SF-36) or better ankle function (FAOS and AOFAS Ankle-Hindfoot score).

#### Satisfaction

The degree of satisfaction was scored by numeric rating scale (NRS), where 0 indicated “very dissatisfied” and 10 “very satisfied.” In addition, participants were asked if they would choose talocrural arthrodesis again.

### Statistical Analysis

Data were analyzed using IBM SPSS Statistics 25. Statistical significance was set at *P* < .05. Data were checked for normality by performing Shapiro-Wilk’s tests and visual inspection of the Q-Q plots.

Patients without preoperative CT were excluded from the study. The baseline characteristics of these patients were compared to the baseline characteristics of the included participants with unpaired *t*-test and chi-square test.

Interrater reliability for the CT OA scale were analyzed by a 2-way random-effect model with absolute agreement. Interrater reliability was assessed on a random subset of 12 CT’s. Interrater reliability scores were interpreted according to the Koch-Landis method, in which kappa (κ) scores can be interpreted as indicating slight agreement (*k* = .01-.2), fair agreement (*k* = .21-.40), moderate agreement (*k* = .41-.60), substantial agreement (*k* = .61-.80), and excellent agreement (*k* = .81-1.00).^19^

To assess differences in OA between preoperative and follow-up CT’s for each adjacent joint, 3 paired *t*-tests were performed per joint with Bonferroni-Holm adjustment to correct for multiple testing for each adjacent joint.

Per adjacent joint, participants were grouped based on pre-existing OA. The cut-off value for pre-existing arthritis was set by ROC analysis and Youden index. In this analysis, the optimal cut-off value for OA ankle scale is set by relating OA ankle scores to the observers’ assessment of OA being present or not present in the joint. The ROC analysis calculates sensitivity and specificity scores for all possible cut-off values.^20^ Youden index is calculated by “sensitivity + specificity-1.” The highest Youden index indicates the optimal cut-off value, that is, optimal balance between sensitivity and specificity.^21^

To investigate increase in OA per group, paired *t*-tests were performed in case of normal distribution, in case of nonnormal distribution, Wilcoxon signed-rank test were done.

To assess whether OA development is associated with time after talocrural arthrodesis, multiple regression analysis was performed for each joint, which were adjusted for age. Also, multiple regression analysis with age adjustment was used to investigate associations between degree of OA at follow-up and patient-reported outcomes (SF-36, FAOS, AOFAS Ankle-Hindfoot score, and satisfaction).

The study was approved by the Erasmus MC Medical Ethical Committee (MEC-2018-153). No funding was received, and the authors have no conflicts of interest to declare.

## Results

### General Characteristics of Study Population

**Figure 3.**
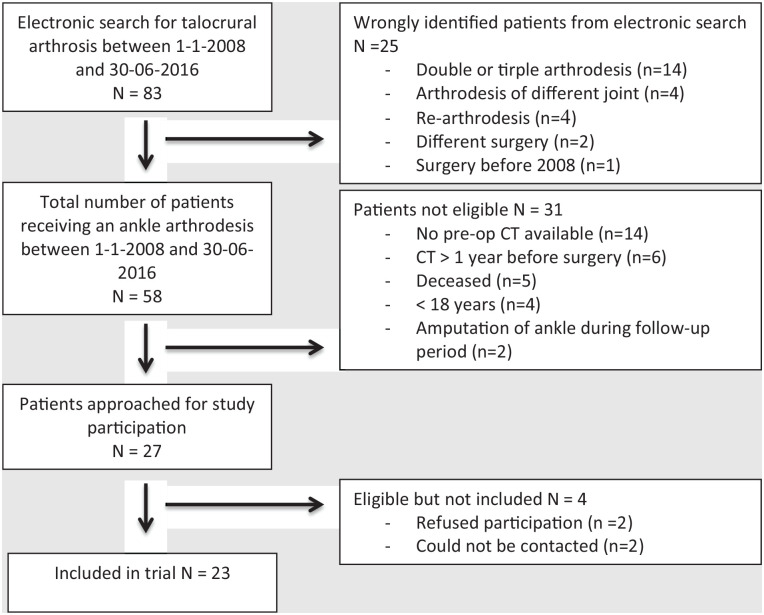
Flowchart of patient selection. Abbreviation: CT, computed tomography.

The electronic search for eligible participants resulted in 83 potentially eligible participants. After screening, 27 eligible patients remained. These patients were approached to participate in the study. Eventually, 23 patients agreed to participate and were included in the study. See Figure 3 for a flow-chart with exclusion reasons for the other patients.

Fourteen patients were excluded because of missing preoperative CT. Baseline characteristics were compared between included patients and patients with missing preoperative CT to investigate possible selection bias. The characteristics age, BMI, operated side, type of OA, and operative technique were not significantly different between patients with and without CT. Time since surgery and sex were significantly different between the groups. Table 1 shows the baseline characteristics of the included participants and characteristics of patients that were excluded due to missing preoperative CT.

**Table 1.** Characteristics of Included Patients and Patients Excluded Due to Missing Preoperative CT.

Variable	Included patients (n = 23)	Excluded patients due to missing CT (n = 14)	*P* value
Average follow-up period in years (SD)	7 (2)	9 (2)	.005*
Average age at arthrodesis in years (SD)	52 (15)	52 (17)	1.0
Average BMI (SD)	28 (4)	28 (6)	.9
Men	10 (44)	11 (79)	.03*
Talocrural arthrodesis at right side	13 (57)	7 (50)	.7
Type of osteoarthritis			.4
Primary	3 (13)	4 (31)	
Secondary	20 (87)	10 (69)	
Operative technique			0.8
Open arthrodesis	4 (17)	3 (21)	
Arthroscopic arthrodesis	19 (83)	11 (79)	

Data are absolute numbers (%) unless otherwise indicated.

Abbreviations: SD, standard deviation; CT, computed tomography; BMI, body mass index.

* Significantly different with *P* < .05.

Nine participants reported problems of the contralateral ankle at follow-up. Three participants had a talocrural arthrodesis, 3 participants reported persistent pain of the ankle after a fracture or trauma, 1 participant had Achilles tendon lengthening, 1 participant had OA in the ankle joint, and 1 participant reported mild ankle complaints.

The 3 participants with a contralateral arthrodesis were excluded from the analysis. Furthermore, 1 participant had an ipsilateral subtalar arthrodesis during follow-up period, and 1 pre-operative CT did not include the calcaneocuboid joint. Therefore, degree of OA could not be measured on these CTs.

### Interrater Reliability for Degree of OA

Interrater reliability was excellent for all features separately and for the overall score, sclerosis *k* = 0.86 (95% confidence interval [CI]: [0.6, 0.9]); cysts *k* = 0.87 (95% CI: [0.7, 0.9]); joint space narrowing *k* = 0.82 (95% CI: [0.6, 0.9]); osteophytes *k* = 0.94 (95% CI: [0.9, 1.0]); overall score *k* = 0.94 (95% CI: [0.9, 1.0]).

### Degree of OA in Adjacent Joints

Overall, the CT OA scale scores were significantly higher for the adjacent joints of the operated ankle at follow-up, compared to preoperative situation and compared to these joints in the control feet. The CT OA scale scores between the preoperative CT and follow-up CT in the control group were not significantly different for any of the adjacent joints, see Table 2.

**Table 2.** CT OA Scale Score in Adjacent Joints Before and After Ankle Arthrodesis.

Joint	Preoperative operated	Follow-up operated	*P* value
Subtalar joint (n = 22)^a^	4.7 (3.0)	7.2 (2.4)	<.001*
Talonavicular joint (n = 23)	3.1 (1.9)	4.8 (2.2)	.003*
Calcaneocuboid joint (n = 22)^b^	2.1 (1.6)	4.1 (1.7)	<.001*
	Preoperative operated	Follow-up control	
Subtalar joint (n = 20)^c^	4.4 (3.0)	3.1 (2.5)	.3
Talonavicular joint (n = 20)^c^	2.9 (1.7)	2.5 (2.0)	1.0
Calcaneocuboid joint (n = 19)^b,c^	2.1 (1.7)	1.7 (2.1)	1.0
	Follow-up operated	Follow-up control	
Subtalar joint (n = 19)^a,c^	7.0 (2.5)	3.2 (2.5)	<.001*
Talonavicular joint (n = 20)^c^	4.9 (2.3)	2.5 (2.0)	.006*
Calcaneocuboid joint (n = 20)^c^	4.2 (1.6)	1.6 (2.0)	<.001*

Data are mean (standard deviation).

Abbreviations: CT, computed tomography; OA, osteoarthritis.

* Significantly different with *P* < .05.

a Data of 1 patient missing due to ipsilateral subtalar arthrodesis during follow-up.

b Data of 1 patient missing due to missing calcaneocuboid joint on preoperative CT.

c Data of 3 patients missing due to arthrodesis of the control ankle.

### Pre-Existing OA

Optimal cut-off value for OA ankle scale to discriminate between joints with pre-existing OA and without pre-existing OA was 3.5. For this cut-off value, the sensitivity and specificity were 0.9 and the Youden index was 0.8.

Table 3 shows the changes in OA in adjacent joints for joints with pre-existing OA and without pre-existing OA. Overall, progression of OA was found in all adjacent joints without pre-existing OA. In adjacent joints with pre-existing OA, progression was only found in the subtalar joint. At follow-up, OA was present in 22 participants (96%) in the subtalar joint, in 15 participants (65%) in the talonavicular joint, and in 14 participants (61%) in the calcaneocuboid joint.

**Table 3.** Changes in CT OA Scale Score for Adjacent Joints With Pre-Existing OA and Without Pre-Existing OA.

Variable	Preoperative	Follow-up	*P* value
Subtalar joint			
Pre-existing OA (n = 14)	5 (4-8)	8 (6-9)	.01*
No pre-existing OA (n = 9)	2 (1-3)	5 (5-8)	.01*
Talonavicular joint			
Pre-existing OA (n = 9)	5 (4-6)	5 (4-7)	.6
No pre-existing OA (n = 14)	2 (1-2)	4 (3-6)	.002*
Calcaneocuboid joint			
Pre-existing OA (n = 3)	5 (4-6)	6 (5-7)	.2
No pre-existing OA (n = 19)	2 (1-3)	4 (2-5)	.001*

Data are median (interquartile range).

Abbreviations: CT, computed tomography; OA, osteoarthritis.

* Significantly different with *P* < .05.

### Patient-Reported Outcomes and Correlations With OA

No significant associations were found between length of follow-up and difference in degree of OA. The SF-36 scores at follow-up are presented in Table 4. For the SF-36, the only domain that was correlated to degree of OA was limitations due to emotional problems. Age-adjusted regression analysis showed significant negative correlations between limitation due to emotional problems and degree of OA at follow-up for the subtalar (*r* = −.6, *P* = .001) and talonavicular joints (*r* = −.6, *P* = .001).

**Table 4.** SF-36 Scores at Follow-Up.

Variable	Mean (SD)
Physical functioning	54.8 (26.0)
Role limitations due to physical health	56.5 (40.7)
Role limitations due to emotional problems	87.0 (34.4)
Energy/fatigue	65.4 (17.6)
Emotional well-being	80.3 (15.7)
Social functioning	78.3 (22.4)
Pain	56.3 (29.0)
General health	56.3 (23.3)
Health change	45.7 (22.2)

Abbreviation: SD, standard deviation.

The average FAOS at follow-up was 53 (SD = 20). The average AOFAS Ankle-hindfoot score at follow-up was 58 (SD = 24). No significant associations were found between scores of the FAOS or AOFAS Ankle-Hindfoot score and the degree of OA of the adjacent joints at follow-up, corrected for age.

### Patient Satisfaction

The median NRS satisfaction score was 8 (interquartile range: 7-9). Most participants indicated that they would probably (17.4%) or definitely (69.6%) have the surgery again if they would be asked to choose again. No significant associations were found between satisfaction rate and degree of OA for any of the adjacent joints at follow-up.

## Discussion

The overall results of this study showed that the talonavicular, calcaneocuboid, and subtalar joints all showed progression of OA after talocrural arthrodesis. Degrees of OA were not significantly different between controls and preoperative OA scores. Based on these results, it seems that OA is a result of talocrural arthrodesis.

Coester et al^7^ compared the degree of OA in adjacent joints between the talocrural side and contralateral side after 22 years of follow-up. In line with the results found in our study, they reported that OA scores were higher at the talocrural side compared to the contralateral side. However, this study had no preoperative imaging.^7^

The studies of Hendrickx et al,^8^ Zwipp et al,^6^ Gaedke et al,^9^ and Jones et al^10^ investigated OA before and after talocrural arthrodesis based on radiographs. The cohorts of these studies were very similar to our cohort with average age ranging from 47 to 61 years, and lengths of follow-up between 5 and 10 years and traumatic OA as primary cause for talocrural arthrodesis. Hendrick et al^8^ reported mild increase in OA in adjacent joints after talocrural arthrodesis. Zwipp et al^6^ reported development of OA in 17% of subtalar joints and 11% of talonavicular joints.

Gaedke et al^9^ reported low pre-existing degrees of OA, which increased after talocrural arthrodesis. This is in accordance with the results found in this study, which showed that in participants without pre-existing OA, OA progresses significantly after talocrural arthrodesis.

The study of Jones et al^10^ reported high rates of pre-existing OA, which remained relatively stable during follow-up for the talonavicular joint. Jones et al^10^ reported that 85% of the patients had no change in talonavicular OA during follow-up. Our study also showed that the degree of OA in participants with pre-existing OA remained relatively stable for the talonavicular and calcaneocuboid joints.

For the subtalar joint, Jones et al^10^ reported no change in OA in 69% of the patients, and thus an increase in OA in 31% of the patients. Zwipp et al^6^ also showed progression of preexisting subtalar OA in 30% of the patients. Our study showed progression of OA in participants with pre-existing subtalar joint OA. It therefore seems that preexisting OA in the subtalar joint becomes worse after talocrural arthrodesis.

Overall, the subtalar joint is most affected by OA as 96% of patients have OA in the subtalar joint at follow-up, compared to 65% and 61% for the talonavicular and calcaneocuboid joints.

In this study, patient-reported outcomes were measured at follow-up with SF-36, FAOS, and AOFAS Ankle-Hindfoot score. For most outcomes, there was no association with OA. Other studies could neither find any correlations between SF-36, AOFAS Ankle-Hindfoot score, or pain with degenerative changes in adjacent joints.^2,7-11^ Our results showed that participants reported high satisfaction rates after talocrural arthrodesis, which were also reported in other studies.^7,8,22-24^ Based on the lack of correlations, it seems that increase in radiologically assessed OA does not have a direct impact on patient-reported outcomes and satisfaction, and that therefore clinical impact is limited.

This study has weaknesses. The rate of participants with pre-existing OA was low in our cohort. In absolute numbers, 9 participants had pre-existing OA in the talonavicular joint and 3 in the calcaneocuboid joint. Increase of OA in the adjacent joints may not have been detected due to low statistical power. Low statistical power may also explain why the statistically significant increase in OA was not associated with length of follow-up. Furthermore, selection bias may have occurred due to the relatively large group of patients that were excluded due to missing preoperative CT but seems to be limited as baseline characteristics between the groups were comparable. There were some missing data in our cohort. One participant received a subtalar arthrodesis during the follow-up, and therefore, the degree of OA could not be assessed. This probably led to a slight underestimation of the degree of OA at follow-up in the subtalar joint of the operated ankle. Three control feet were excluded because they had undergone talocrural arthrodesis and could therefore not serve as controls. This may have resulted in an underestimation of degree of OA in the control group. In addition, this is a retrospective cohort without a power calculation. Therefore, nonsignificant findings and lack of significant associations might be the result of low power. However, despite the above limitations, this is the first study with preoperative CT and postoperative CT which is a strong feature of this study. To draw more firm conclusions, future studies should include higher number of patients. Also for future studies weight-bearing CT should be considered as joint space may decrease at weight-bearing, which is missed with standard CT.^25^

## Conclusion

This study showed that progression of OA in the adjacent tarsal joints is a consequence of talocrural arthrodesis. Especially adjacent joints without pre-existing OA develop OA after talocrural arthrodesis. The subtalar joint is the most affected by OA with high preoperative OA scores and progression of OA after talocrural arthrodesis. Progression of OA in adjacent joints does not seem to affect patient-reported outcome measures or satisfaction.
